# Metabolic Changes in Larvae of Predator *Chrysopa sinica* Fed on Azadirachtin-Treated *Plutella xylostella* Larvae

**DOI:** 10.3390/metabo12020158

**Published:** 2022-02-08

**Authors:** Peiwen Zhang, You Zhou, Deqiang Qin, Jianjun Chen, Zhixiang Zhang

**Affiliations:** 1 Key Laboratory of Natural Pesticide and Chemical Biology, Ministry of Education, South China Agricultural University, Guangzhou 510642, China; peiwen.zhang@ufl.edu (P.Z.); 20181023002@stu.scau.edu.cn (D.Q.); 2 Mid-Florida Research and Education Center, Department of Environmental Horticulture, Institute of Food and Agricultural Sciences, University of Florida, Apopka, FL 32703, USA; 3 College of Biology and Food Engineering, Chongqing Three Gorges University, Chongqing 404100, China; youzhou@sanxiau.edu.cn

**Keywords:** azadirachtin, biological control, *Chrysopa sinica*, metabolomics, *Plutella xylostella*

## Abstract

Biological control is a key component of integrated pest management (IPM). To suppress pests in a certain threshold, chemical control is used in combination with biological and other control methods. An essential premise for using pesticides in IPM is to ascertain their compatibility with beneficial insects. *Chrysopa sinica* (Neuroptera: Chrysopidae) is an important predator of various pests and used for pest management. This study was intended to analyze metabolic changes in *C. sinica* larvae after feeding on azadirachtin-treated *Plutella xylostella* (Lepidoptera, Plutellidae) larvae through a non-targeted LC–MS (Liquid chromatography–mass spectrometry) based metabolomics analysis. Results showed that *C. sinica* larvae did not die after consuming *P. xylostella* larvae treated with azadirachtin. However, their pupation and eclosion were adversely affected, resulting in an impairment in the completion of their life cycle. Feeding *C. sinica* larvae with azadirachtin-treated *P. xylostella* larvae affected over 10,000 metabolites across more than 20 pathways, including the metabolism of amino acids, carbohydrates, lipid, cofactors, and vitamins in *C. sinica* larvae, of which changes in amnio acid metabolism were particularly pronounced. A working model was proposed to illustrate differential changes in 20 metabolites related to some amino acid metabolisms. Among them, 15 were markedly reduced and only five were elevated. Our results suggest that azadirachtin application may not be exclusively compatible with the use of the predator *C. sinica* for control of *P. xylostella*. It is recommended that the compatibility should be evaluated not only based on the survival of the predatory insects but also by the metabolic changes and the resultant detrimental effects on their development.

## 1. Introduction

Integrated pest management (IPM) is a coordinated process using multiple methods, such as biological, chemical, cultural, mechanical, physical, and pest resistant or tolerant varieties for optimizing control of pests in an ecologically and economically sound manner [1]. Among them, chemical control and biological control are two common methods used either separately or in combination for managing insect pests during crop production. However, the use of pesticides may have adverse effects on non-target organisms, including predatory insects [2]. In order to minimize these problems and maintain sustainable control of insect pests, botanical pesticides are considered to be attractive alternatives for pest management. Compared with traditional synthetic insecticides, botanical pesticides have better eco-toxicological properties, including low toxicity, rapid degradation, and little impact on the environment, which makes them a suitable choice for pest control [3,4].

Azadirachtin is the main active ingredient in neem (*Azadirachta indica*), which shows insecticidal, fungicidal, and bactericidal activities [3,5]. Azadirachtin as an insecticidal ingredient has been reported to effectively control more than 400 species of insects [6]. This compound displays multiple effects on insects and can be used as an insect repellent, antifeedant, growth retardant, molting inhibitor, and oviposition deterrent [5,6,7]. Azadirachtin was reported to be selective and would not affect important natural enemies of pests [8]. The application of azadirachtin did not affect the natural enemies of red spider mite, *Mallada desjardinsi* and *Oligota pygmaea* [9]. However, the neem oil cause anomalies on wings and legs of the non-target predator *Podisus nigrispinus*, thereby affecting its growth and causing its death [5]. Azadirachtin significantly affected longevity, number of attacked hosts, and progeny size of females on *Opius concolor* and could slightly reduce the survival rate of emerged *Podisus maculiventris* adults [10,11]. Additionally, azadirachtin was reported to affect searching time, mating behavior, and egg-laying rate of coconut mite predator *Neoseiulus baraki* [12]. Thus, even when botanical pesticides, such as azadirachtin are used in IPM, knowledge about their relative toxicity to and compatibility with predatory insects should be acquired.

The green lacewing, *Chrysopa sinica* Tiedet (Neuroptera: Chrysopidae), is a polyphagous natural enemy attacking several pests on various crops. The lacewing larvae prey on the insect’s eggs, nymphs, larvae, and adults, such as nymphs and adults of Hemiptera, eggs and larvae of Lepidoptera [13,14,15]. They consume pollen, nectar, insect eggs, and younger larvae during its adult stage [14]. Lacewing is commercially produced in many countries for use as a biological agent against a wide range of pests, particularly in China [16]. Thus far, little information is available about azadirachtin effects on *C. sinica* although its effects on other lacewings were reported. There were no obvious effects on lacewing *Chrysoperla carnea* larvae after they were fed azadirachtin treated *Sitotroga cerealella* eggs [10,11]. Azadirachtin was not significantly toxic to eggs and pupae of *C. carnea* but caused a slight reduction in the number of pupae and adults [17]. However, other reports indicated that neem oil products adversely affect growth and development of lacewings *Ceraeochrysa claveri* either directly or indirectly [18,19,20,21]. To appropriately use azadirachtin and *C. sinica* for pest management, it is important to understand their compatibility not only based on the survival but also by their biochemical and metabolic responses. 

Azadirachtin can adversely affect the endocrine system [22], cell division, protein synthesis [23], and different enzymes including NADPH cytochrome reductase, and cholinesterase [24,25]. Azadirachtin was reported to affect genes involved in larval or pupal development, defense responses, carbohydrate metabolism, signal transduction, and chemosensory behavior, as well as proteins implicated in energy metabolism, cytoskeletal structure, transcription and translation, and hormonal regulation in *Drosophila* larvae [26,27]. Similar effects were reported in *Plodia interpunctella* [28], *Glyphodes pyloalis* larvae [29], *Ostrinia furnacalis* larvae [30], *Hyphantria cunea* larvae [31], and *Helicoverpa armigera* [32]. Metabolomics is an important analytical tool of systems biology, which can identify all detectable metabolites in a biological system [33]. It is used to reveal endogenous metabolite changes that are caused by drug toxicity, disease process, or gene function [34,35,36,37]. Liquid chromatographic and gas chromatography-mass spectrometry (LC–MS and GC–MS) has been increasingly used to document metabolomic profiles of insect pests [24,38,39,40,41]. Recently, metabolic changes of *Bactrocera dorsalis* larvae [24] and *Helicoverpa armigera* [32] resulting from the treatment of azadirachtin have been reported, which provide more detailed information on azadirachtin actions to respective insect pests. Thus far, there has no metabolomic analysis of azadirachtin effects on *C. sinica*, especially after consumption of azadirachtin-treated preys. 

The objectives of this study were to analyze metabolic changes in *C. sinica* larvae after feeding *Plutella xylostella* (Lepidoptera, Plutellidae) larvae treated with azadirachtin using ultra high performance liquid chromatograph coupled with tandem mass spectrometry (UHPLC–MS/MS), identify main metabolic pathways altered by the consumption of azadirachtin-treated prey, outline key components underlying the detrimental effects, and address likely problems for the use of azadirachtin and *C. sinica* in control of *P. xylostella* in crop production.

## 2. Results

### 2.1. Azadirachtin Activities against C. sinica

**Figure 1:**
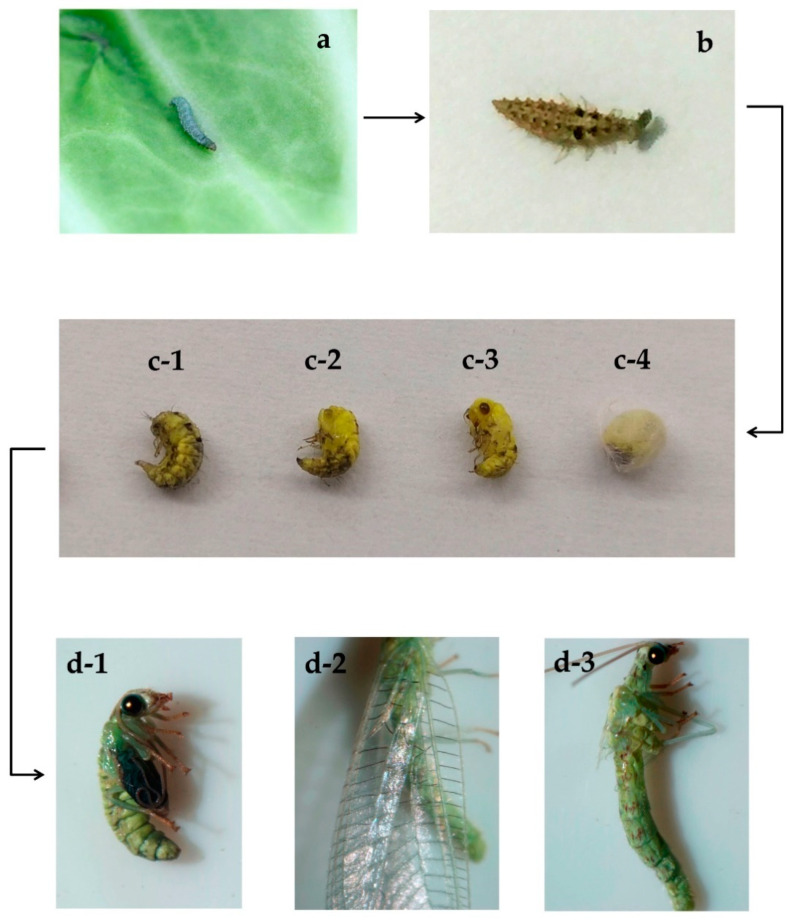
The process of monitoring *P. xylostella* larvae feeding on cabbage leaves and *C. sinica* larvae ingesting azadirachtin-treated *P. xylostella* larvae and subsequently their pupation and eclosion. A larva feeding on a cabbage leaf treated with azadirachtin (**a**). A *C. sinica* larva ingesting a *P. xylostella* larva (**b**). *C. sinica* larvae underwent pupation from curling to the formation of cocoon (**c-1**–**c-4**). The emergence of *C. sinica* adult: a deformed adult (**d-1**), normal adult (**d-2**), and normal adult after removing wings to show normal abdomen (**d-3**).

**Figure 2:**
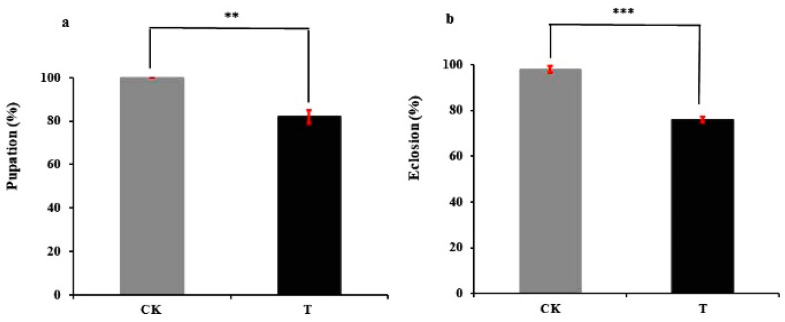
The proportion of mature *C. sinica* larvae underwent pupation (**a**) and eclosion (**b**) after ingesting azadirachtin-treated *P. xylostella* larvae. Data were expressed as the mean ±  S.E. and ** and *** indicate significant differences at *p*  <  0.01 and *p*  <  0.001 levels based on Tukey’s HSD test.

**Figure 3:**
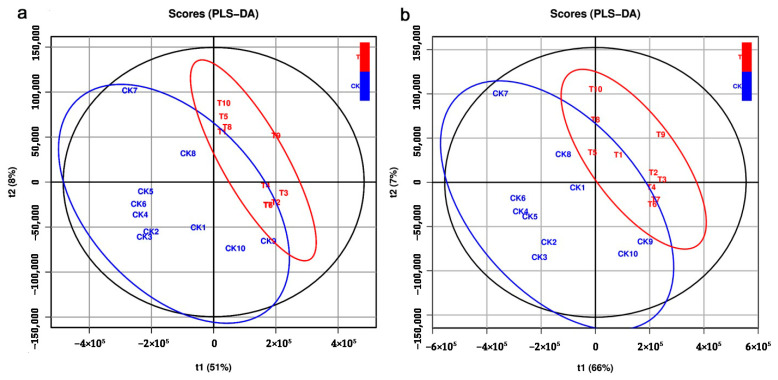
PLS-DA (partial least squares discriminant analysis) score plots derived from (**a**) positive ion mode (ESI+) and (**b**) negative ion mode (ESI−) in LC–MS metabolite profiles of *C. sinica* larvae.

**Figure 4:**
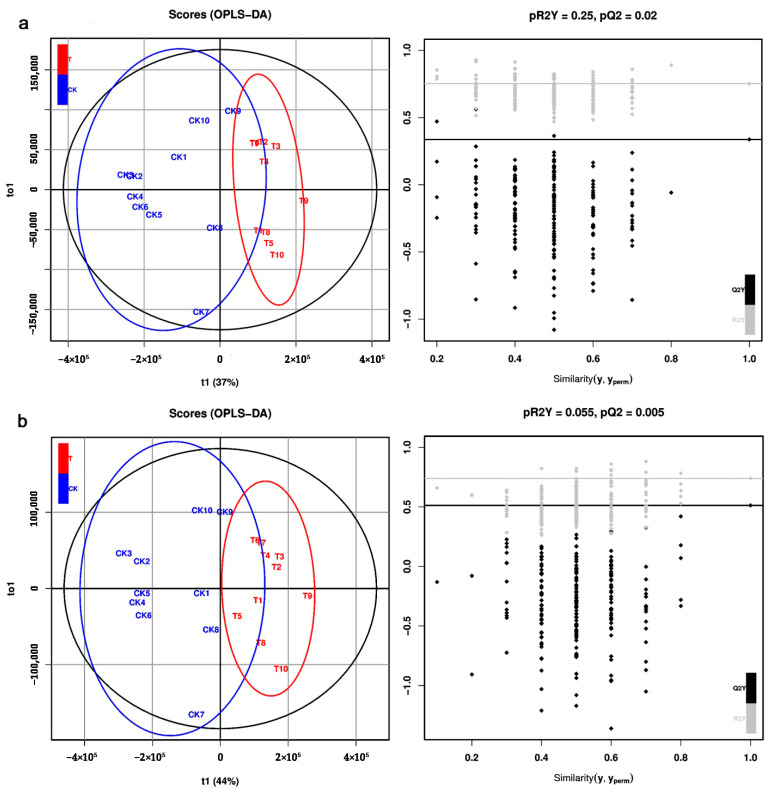
OPLS-DA (partial least squares discriminant analysis) score plots (**left**) with corresponding permutation test plots (**right**) derived from (**a**) positive ion mode (ESI+) and (**b**) negative ion mode (ESI−) in LC–MS metabolite profiles of *C. sinica* larvae.

The larvae of *C. sinica* fed with *P. xylostella* larvae that consumed azadirachtin-treated leaves (T) showed no significant growth differences compared to those fed *P. xylostella* control (CK) as there were no larval mortalities between T and CK before pupation (data not shown). After stopping preying, mature larvae began to pupate. The head and tail of the larvae gradually curled together, and the tail drew silk to make cocoons (Figure 1(c-4)). However, there were 18% of the larvae in the T treatment that could not curl up and could not draw silk to make a cocoon (Figure 1(c-1–c-3)). When touching the larvae with a small brush, they still twisted but died in a few days. After 20 to 30 days, the *C. sinica* adults emerged from the pupae. In the T treatment, 24% of adults were deformed as they were unable to extend their wings and/or had malformed abdomen (Figure 1(d-1)), which were regarded as the failure to eclosion. Adults with fully extended wings and no growth defects were considered as successful eclosion (Figure 1(d-2,d-3)). Thus, the pupation and eclosion of *C. sinica* larvae were significantly affected by T treatment. As shown in Figure 2, the proportion of larvae undergoing pupation from T treatment was significantly lower at 82.00  ±  3.06% comparted to 100.00% in the CK (*p * <  0.01), and their eclosion was 76.00  ±  1.15% in the T treatment against 98.00  ±  1.48% of CK (*p*  <  0.001).

### 2.2. Metabolic Profiles Analyzed by LC–MS

The unsupervised PCA was used to check the quality of the data from the LC–MS analyses. In ESI+ mode, the PC1 and PC2 explained 30.9% and 11.4% of the total variance of all samples. In ESI− mode, the PC1 and PC2 explained 33.9% and 10.3% of the total variance. The supervised PLS-DA was performed to identify the metabolites responsible for the separation between CK and azadirachtin treatments (T). Results showed that in the ESI+ mode, the R^2^X, R^2^Y, Q^2^Y, and RMSEE values in the PLS-DA model were 0.59, 0.753, 0.369, and 0.269 (Figure 3a), respectively; in the ESI− mode, the R^2^X, R^2^Y, Q^2^Y, and RMSEE values in the PLS-DA model were 0.728, 0.74, 0.536, and 0.276 (Figure 3b), respectively. Based on the OPLS-DA model, the R^2^X, R^2^Y, Q^2^Y, and RMSEE values in the ESI+ mode were 0.59, 0.753, 0.337, and 0.269 (Figure 4a), respectively. In the ESI− mode, the R^2^X, R^2^Y, Q^2^Y, and RMSEE values in the ESI− mode were 0.728, 0.74, 0.512 and 0.276 (Figure 4b), respectively. 

### 2.3. Changes in Metabolites and Metabolic Pathways of Differentially Abundant Metabolites 

**Figure 5:**
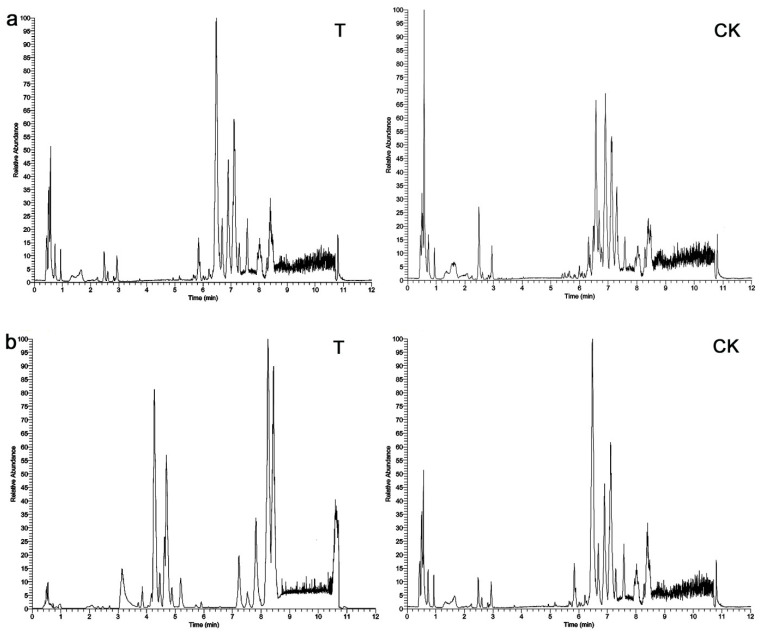
Representative total ion current (TIC) chromatograms of *C. sinica* larvae tissue extracts obtained from (**a**) positive ion mode (ESI+) and (**b**) negative ion mode (ESI−) in LC–MS. Left plots were treatment samples (T), and right plots were control samples (CK).

Representative LC–MS total ion chromatograms (TICs) of *C. sinica* larvae tissue samples are shown in Figure 5. The shape and quantity of peaks between the T and CK treatments varied greatly. Approximately 13,672 and 10,947 metabolite peaks were deconvoluted in ESI+ and ESI− modes of LC–MS, respectively. The ESI+ usually detects N, O, and S-containing species and also some specific hydrocarbons, such as isoprene, terpenes, and aromatics as protonated neutral MH+, whereas the ESI− detects acid including carboxylic acids RCOOH and inorganic acids and hydrosulfides as deprotonated neutral [M-H]^−^ [42]. A total of 3210 and 2026 remaining peaks in ESI+ and ESI− modes in LC–MS were further annotated using references in existing databases, respectively. After the exogenous compounds in LC–MS were removed, the differentially abundant metabolites were selected according to the VIP values from the OPLS-DA model (VIP ≥ 1) and the corrected *p* values from *t*-test (*p* < 0.05). There were 778 compounds in the ESI+ model, of which 357 were upregulated and 421 were downregulated. In ESI− mode, there were 391 compounds: 180 were upregulated and 211 were downregulated. 

**Figure 6:**
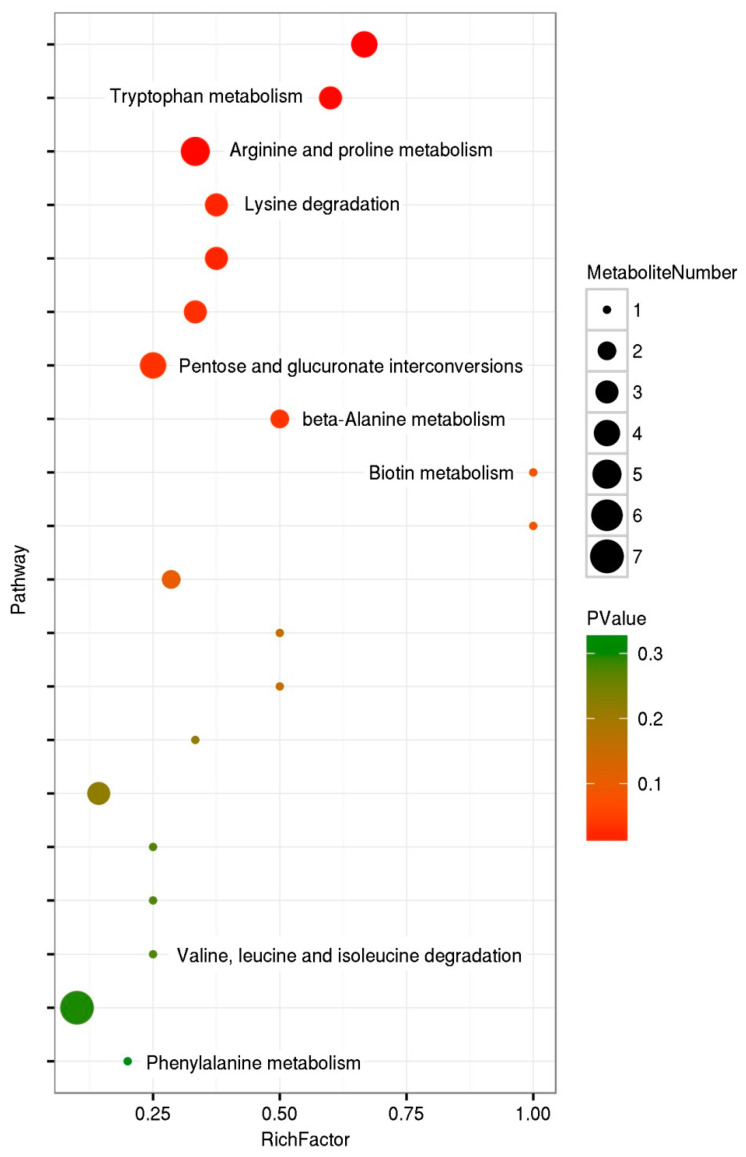
Metabolome map of significant metabolic pathways in *C. sinica* larvae affected by the ingestion of azadirachtin-treated *P. xylostella* larvae (pathway enrichment). Rich factor refers to the ratio of the number of annotated to this pathway in the differential metabolites to the number of annotated to this pathway in all metabolites. A larger rich factor indicates a higher degree of enrichment. *p* values range from 0 to 1, the closer to 0, the more significance of the enrichment.

Table 1 shows some related metabolic pathways with representative differentially abundant metabolites and their upregulation and downregulation. Amino acids, carbohydrates, bile, lipids, membrane transports, cofactors and vitamins were primary metabolites that were affected in *C. sinica* larvae after ingestion of azadirachtin-treated *P. xylostella* larvae. The enrichment of pathways is presented in Figure 6. Rich factor refers to the ratio of the numbers of differentially abundant metabolites annotated in this pathway to the numbers of all metabolites annotated to the same pathway. The greater the rich factor, the greater the pathway enrichment. The *p* value is another parameter for enrichment with a range from 0 to 1, the closer to 0, the more significance of the enrichment. As shown in Figure 6, biotin metabolism was significantly enriched, but the numbers of metabolites were much lower. Tryptophan metabolism was also significantly enriched with relatively higher metabolite numbers. Arginine and proline metabolism had a Rich factor of 0.33 with rather higher metabolite numbers. Lysine degradation had comparable Rich factors and also higher metabolite numbers. Additionally, beta-alanine metabolism was significantly relevant pathway of *C. sinica* larvae that were affected by ingestion of azadirachtin-treated *P. xylostella* larvae.

## 3. Discussion

### 3.1. Metabolite Changes Adversely Affected C. sinica Development 

The present study showed that no mortality occurred in *C. sinica* larvae after consuming azadirachtin-treated *P. xylostella* larvae. However, their pupation and eclosion were significantly affected. Compared with the control treatment, 18% and 24% of the mature larvae were unable to perform pupation and eclosion, respectively (Figure 1 and Figure 2). These results suggest that *C. sinica* larvae were able to obtain needed nutrients through digesting *P. xylostella* larvae to sustain their growth and even tolerate ingested azadirachtin, but the azadirachtin adversely affect *C. sinica* metabolism. As shown in Table 1 and Figure 6, over 10,000 metabolites across more than 20 pathways, including the metabolism of carbohydrates, lipid amino acids, vitamins and their cofactors, and amino acids were changed in *C. sinica* larvae. 

Amino acids are fundamental for synthesizing proteins and phospholipids, energy production, and involved in morphogenetic processes. In this study, lysine degradation, tryptophan metabolism, phenylalanine metabolism, arginine and proline metabolism, valine, leucine, and isoleucine degradation were substantially down-regulated (Table 1). The reduced metabolism of these amino acids could significantly impair *C. sinica* growth and development. 

The carbohydrate metabolism is essential for cellular energy balance and for the biosynthesis of new cellular building blocks [43]. In this study, 10 carbohydrate pathways were down-regulated, and eight pathways were upregulated in both ESI+ and ESI− modes (Table 1). Among them, succinic acid was a differentially enriched metabolite in tricarboxylic acid (TCA) cycles. The TCA cycle, known as the citric acid cycle, has an important function that involves the intermediate compounds for the synthesis of amino acids and fatty acids and the formation of large quantities of adenosine triphosphate (ATP) that provides energy for various biological processes [24]. The downregulation of succinic acids of carbohydrate metabolites could cause a shortage of intermediate compounds and energy in azadirachtin-ingested *C. sinica* larvae, impairing their growth and development. Additionally, the amino sugar and nucleotide sugar metabolism pathway was enriched in azadirachtin-ingested *C. sinica* larvae. D-glycerate 3-phosphate was also among the differentially enriched metabolites in glycolysis/gluconeogenesis pathway. Glycolysis and gluconeogenesis are metabolic processes responsible for glucose degradation or glucose synthesis, respectively [44]. 

Ingestion of azadirachtin-treated *P. xylostella* larvae also affected the metabolism of vitamins and their cofactor in *C. sinica* larvae. Biotin is an essential substance for insects and affects the development of advanced larvae and pupae. The biotin metabolism pathway was significantly enriched in *C. sinica* larvae. The downregulation of this metabolite could adversely affect normal metabolism and *C. sinica* development.

Glycolysis is the main metabolic pathway of carbohydrates, such as galactose and fructose [44]. As an intermediate in both glycolysis and gluconeogenesis, the change in the relative content of D-glycerate 3-phosphate could also affect the generation of intermediate compounds and energy to maintain normal biological processes. Furthermore, the pathways of pentose and glucuronate interconversions, C-5 branched dibasic acid metabolism, ascorbate, and aldarate metabolism were enriched in azadirachtin-ingested *C. sinica*. Such a series of changes in carbohydrate metabolisms would affect the energy supply of *C. sinica* larvae, thus their development. Lipids are of vital importance to insects as energy sources and substrates for embryogenesis and development, pupation, metamorphosis, and other activities [45]. They are important components of insect cell membrane and also precursors of many insect pheromones [45]. Azadirachtin could influence the quantity and relative composition of fatty acids [30]. In the present study, 11 lipids or lipid-like metabolites were found to be differentially abundant, of which seven were upregulated, and four were down-regulated. The downregulated included palmitoleic acid, linolenic acid, 9-hydeoxyoctadecadienoic acid (9-OxoODE), and arachidic acid. Linolenic acid plays an important role in insect reproduction as it is a key constitute of oocyte dry mass and the major energy source for embryo development. The linolenic acid has been documented to be required for developing *Heliothines subflexa* [46]. The reduction in metabolism of linolenic acid in azadirachtin-ingested *C. sinica* larvae may potentially affect the pupation and eclosion, which required further confirmation. 

### 3.2. Key Metabolic Pathways Affected by Feeding on Azadirachtin-Treated P. xylostella Larvae 

**Figure 7:**
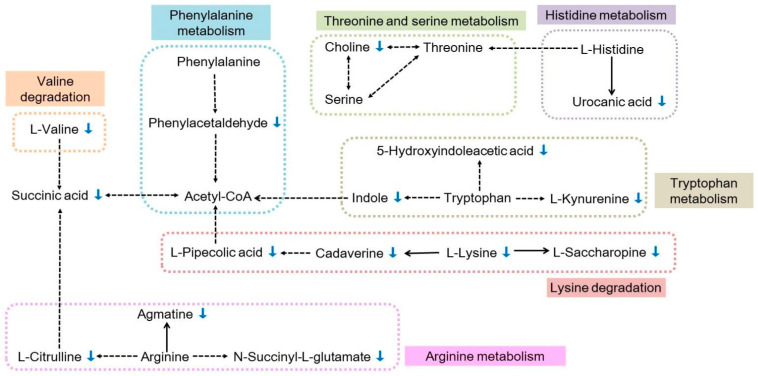
Metabolic pathways related to amino acid metabolism in *C. sinica* larvae affected by the ingestion of azadirachtin-treated *P. xylostella* larvae. The blue arrows indicate decreased metabolites.

The aforementioned analyses provide an overall spectrum of metabolite changes in *C. sinica* after ingestion of azadirachtin-treated *P. xylostella* larvae. The next question would be which metabolic pathways might be specifically implicated in the reduced percentages in pupation and eclosion *C. sinica*. Through the KEGG pathway analysis of the differentially abundant metabolites, the disturbed metabolic pathways caused by the consumption of azadirachtin-treated *P. xylostella* larvae were analyzed. A working model was constructed using the reference map deposited in the KEGG database (Figure 7). It was noticed that 20 differential metabolites were related to amino acid metabolic pathways. Among them, 15 metabolites were down-regulated. These results could imply that the ingestion of azadirachtin-treated *P. xylostella* larvae might result in the impairment in hydrolyzing proteins in *C. sinica* resulting in an insufficient supply of amino acids or directly affect amino acid metabolisms. Amino acids, particularly the essential ones are fundamental for insect growth and development [47]. A distinct biochemical characteristic of insects is their higher levels of free amino acids in the hemolymph [48], and the likely utilization of the free amino acids as silk protein synthesis to produce cocoon [49]. Furthermore, the eclosion is controlled by three peptide hormones: eclosion hormone, ecdysis-triggering hormone, and crustacean cardioactive peptide [50,51]. The reduction of free amino acids could hamper both pupation and eclosion of *C. sinica*.

As shown in Figure 7, a majority of essential amino acid metabolisms were downregulated. A limited supply of tryptophan resulted in the decrease in the contents of indole, L-kynurenine, and 5-hydroxyindoleacetic acid. The downregulation of these compounds could limit the biosynthesis of acetyl-CoA, affecting the TCA cycle. It is worth mentioning that metabolites of the kynurenic pathway generally reach to peak concentrations in insects during pupation [52,53]. The reduced availability of kynurenine could impair pupation in *C. sinica*. Limited availability of lysine caused reduced biosynthesis of cadaverine and L-pipecolic acid. The downregulation of cadaverine also affected acetyl-CoA biosynthesis, subsequently affecting the TCA cycle. In the arginine metabolism pathway, low arginine in cells resulted in reduced synthesis of N-succinyl-L-glutamate, L-citrulline, and agmatine. Arginine can be converted to proline. Proline was a major substrate used in insect flight metabolism, which is known as the fuel of insect flight [54]. Choline was downregulated, which could indirectly affect the metabolism of serine and threonine. Pyruvate, an indirect derivative of choline, also from glycolysis is regarded as a key metabolite producing valine [55]. Valine also affects acetyl-CoA. L-valine was significantly low in *C. sinica*. Taken together, at the time of requiring higher levels of free amino acids in the hemolymph, the reduced amino acid metabolisms, particularly the essential ones significantly affected the pupation and eclosion of *C. sinica*. 

### 3.3. Precautions When Azadirachtin and C. sinica Are Used in IPM

The changes in a wide range of metabolites in *C. sinica* larvae suggest that the action mode of azadirachtin is multifaceted with multiple biological targets. Thus, precautions should be taken when azadirachtin and *C. sinica* are to be used in IPM. Although this study was mainly focused on metabolites without further analysis of the biological influence of these molecular alterations on natural enemies, our data did document that *C. sinica* larvae after ingestion of larvae of *P. xylostella* treated with azadirachtin at 2.00 mg/L significantly reduced pupation and eclosion. Such adverse effects were related to the substantial changes in metabolomic profile of *C. sinica*. As mentioned previously, the premise for the use of pesticides, even botanical ones, in IPM is ascertaining their compatibility with beneficial predators. Our study showed azadirachtin and *C. sinica* are not compatible, which primarily agrees with the results of *C. claveri* in responses to azadirachtin [18,19,20,21]. Commercially, azadirachtin has been applied at much higher concentrations ranging from 5 to 100 mg/L [56]. Thus, detrimental effects to beneficial insects could be even severer. However, this does not exclude the use of azadirachtin and *C. sinica* at different time periods. Nevertheless, our study has raised the compatibility question between the two control tactics. Further studies are needed to identify specific mechanisms underlying the reduced pupation and eclosion of *C. sinica* larvae, effects on mating and oviposition of adults, and the relationship between azadirachtin concentrations and metabolite changes as well as threshold concentrations of azadirachtin to *C. sinica* for potentially better use of the two tactics for pest management.

## 4. Materials and Methods

### 4.1. Chrysopa sinica and Plutella xylostella

*Chrysopa sinica* and *Plutella xylostella* were raised in the insect rearing facility of the Key Laboratory of Natural Pesticide and Chemical Biology, Ministry of Education, South China Agricultural University, Guangzhou, China. *C. sinica* larvae were fed larvae of *P. xylostella*, and the adults were fed on a diet of *P. xylostella* larvae, along with 15% honey water, and yeast powder. The larvae of *P. xylostella* were fed leaves of the host cabbage (*Brassica oleracea* L.) plants, and the adults were fed 15% honey water. The temperature in the insect rearing facility was 25 ± 1 °C, relative humidity was 60 ± 5%, and a light-dark cycle of 16 h and 8 h.

### 4.2. Chemical Reagents and Instruments 

Reagents including methanol, acetonitrile, ammonium acetate, ammonium hydroxide, and formic acid were purchased from CNW Technologies (ANPEL Laboratory Technologies, Inc. Shanghai, China). The internal standards of 2-chloro-L-phenylalanine was purchased from Shanghai Hengbai Biotech Co., Ltd. (Shanghai, China), and azadirachtin (>90%) were provided by Associate Professor Yongqing Tian at the South China Agricultural University.

Major instruments used in this study included ultra-high performance liquid chromatography (UHPLC) (Agilent Technologies, Santa Clara, CA, USA) coupled with Q Exactive Focus mass spectrometer (MS) (Thermo Fisher Scientific, Waltham, MA, USA), centrifuge, scales, grinding mill, water purification, ultrasound instrument, and column (ACQUITY UPLC HSS T3 1.7 μm, 2.1 × 100 mm).

### 4.3. Experimental Procedures and Samples Collection

Cabbage plants were singly grown in 15-cm pots filled with a peat-based potting substrate. When plants were at a stage of 10 leaves, 20 plants with a uniform growth size were randomly selected. The third instar larvae of *P. xylostella* were placed on cabbage leaves, 50 larvae per plant. Azadirachtin stock solution (10,000 μg/mL) was made by dissolving it in acetone. The stock solution was diluted with water resulting in a working solution of azadirachtin at 2.00 mg/L, which was sprayed on leaves of 10 plants (5 mL per plant) as azadirachtin treatment (T). The other 10 plants were sprayed with the same concentration of acetone (0.02%) in the same volume per plant as control treatment (CK). After the *P. xylostella* larvae had eaten the treated cabbage leaves for 12 h (Figure 1a), the third instar larvae of *C. sinica* were released and fed continuously with *P. xylostella* larvae from T and CK plants for 5 days, respectively (Figure 1b). The experiment was arranged as a complete randomized design with 10 replications. Five days later, more than fifty larvae of *C. sinica* were collected from each plant, quickly frozen in liquid nitrogen, and stored at −80 °C. The stored larvae samples were ground into a fine powder in liquid nitrogen and freeze-dried for 24 h until extraction. 

To monitor the development of *C. sinica*, the pupation and eclosion of *C. sinica* larvae on cabbage plants of the two treatments were observed. After 10 days, when a complete white round cocoon was formed, the pupation was considered complete (Figure 1c). After 20 days, the adults broke out of the cocoon and spread their wings; they were deemed to have completed their emergence (Figure 1d). The experiment was arranged as a randomized complete block design with three replications (three blocks), each block had 50 larvae. The proportion of pupation and eclosion were calculated and analyzed using SPSS software platform (25.0) (IBM Corporation, Somers, NY, USA), and means were separated based on Tukey’s HSD (honestly significant different) test at *p* < 0.01 and *p* < 0.001 levels. 

### 4.4. Metabolites Extraction and Detection 

The ground samples of control (CK) and azadirachtin treatment (T), 100 mg each, 10 replicates per treatment, were placed into Eppendorf tubes, respectively. To each tube was added 300 μL methanol and 20 μL 2-chloro-L-phenylalanine, the samples were vortexed for 30 s and sonicated for 5 min in the ice-water bath. The homogenate and sonicate circles were repeated for 3 times, followed by incubation at −20 °C for 1 h and centrifugation at 13,000 rpm and 4 °C for 15 min. The resulting supernatants were transferred to LC–MS vials and stored at −80 °C until analysis. 

The quality control (QC) sample was prepared by mixing an equal aliquot of the supernatants from all of the samples to analyze the repeatability of samples under the same processing method. In the process of analysis, one quality control sample was inserted every 6–10 test analysis samples to monitor the repeatability of the analysis process. 

The platform for LC–MS analysis consisted of an UHPLC system (1290, Agilent Technologies) with a UPLC HSS T3 column coupled to Q Exactive Orbitrap mass spectrometer (QEO MS) (Thermo Fisher Scientific). The supernatant (200 μL) was taken into the sample bottle (2 mL), respectively.

Mobile phase conditions were set as follows: the mobile phase A was 0.1% formic acid in water for positive, and 5 mmol/L ammonium acetate in water for negative (adjusted the PH value to 9.0 with ammonia), and the mobile phase B was acetonitrile. The elution gradient of the mobile phase was shown in supplementary file, Table S1. 

Mass spectrometry conditions included the use of QEO MS to collect MS and MS/MS data with the electrospray (ESI) source conditions set as follows: spray voltage as 3800 V for positive or −3100 V for negative, capillary temperature 320 °C, sheath gas flow rate as 45 Arb, aux gas flow rate as 15Arb, full MS resolution as 70,000, MS/MS resolution as 17,500, strength of collision energy as 3, collision energy as 20/40/60 eV, scanning scope as 70–1000 m/z, scan rate as 7 Hz.

### 4.5. Data Preprocessing and Multivariate Statistical Analysis 

The original LC–MS data files were converted to the mzML format by using ProteoWizard and processed by R package XCMS (version 3.2), including retention time alignment, peak detection, peak matching, and peak integration. The data were then filtered by the following criterion: sample numbers containing a metabolite were less than 50% of all sample numbers in a group. OSI-SMMS (version 1.0, Dalian Chem Data Solution Information Technology Co. Ltd, Dalian, China) was used for peak annotation after data processing with in-house MS/MS database. 

To initially visualize the differences between different groups of samples, the principal component analysis (PCA) was applied. PCA analysis is an unsupervised multi-dimensional statistical analysis method that describes the characteristics of the original data set by compressing the original data into countless principal components, which can reflect the overall metabolic difference between each group of samples and the size of variation between the group samples. Partial least squares discriminant analysis (PLS-DA), as a supervised multivariate statistical analysis method, was used to distinguish the metabolomics profile of two groups by screening variables correlated to class memberships in which class memberships were coded in matrix form into Y [57]. Orthogonal projection to latent structures-discriminant analysis (OPLS-DA) is an extension of PLS-DA which incorporates an orthogonal signal correction (OSC) filter into a PLS model. The model quality was assessed based on cross-validation and permutation test [58]. The variable importance in projection (VIP) score of OPLS-DA model and *t*-test as a univariate analysis were applied to rank the metabolites that best distinguished the different groups in this study. Those with VIP ≥ 1 and a *p*-value of *t*-test < 0.05 were considered differential metabolites between two groups [59]. 

### 4.6. KEGG Pathway Analysis 

After the metabolites were found, the metabolites were mapped to KEGG metabolic pathways for pathway analysis and enrichment analysis [60]. The main biochemical metabolic pathways and signal transduction pathways in differential metabolites were analyzed in this study. Significantly enriched metabolic pathways or signal transduction pathways in differential metabolites comparing with the whole background were identified through pathway enrichment analysis.

## 5. Conclusions

Azadirachtin as a botanical pesticide has been increasingly used for control of insect pests. This study investigated responses of predator *C. sinica* larvae after ingestion of *P. xylostella* larvae treated with azadirachtin, mainly at the metabolite levels. No mortality occurred in *C. sinica* larvae after consuming azadirachtin-treated *P. xylostella* larvae, but the percentages of pupation and eclosion of *C. sinica* were significantly reduced. Metabolomic analysis showed that azadirachtin has effects on the metabolism of amino acids, carbohydrates, lipid, cofactors and vitamins of *C. sinica* larvae. These effects may impair the growth and development of *C. sinica*, resulting in reduced pupation and eclosion percentages. Our studies for the first time documented substantial metabolite changes in *C. sinica* larvae after ingestion of azadirachtin-treated *P. xylostella* larvae and raise a question about the compatibility between azadirachtin and *C. sinica* in control of insect pests through IPM. 

## Figures and Tables

**Table 1:** Differentially abundant metabolites identified in ESI+ and ESI− modes of LC–MS analysis of extracts derived from *C. sinica* larvae fed azadirachtin-treated *P. xylostella* larvae in contrast to those from the control treatment.

Mode	KEGG Class	Pathway	Regulation	*p*-Value ^†^	VIP ^‡^	Name	Formula	M/Z ^§^
**ESI+**	Amino acid metabolism	Glycine, serine, and threonine metabolism	down	6.86 × 10^−3^	11.98	Choline	C_5_H_13_NO	104.1068832
		Arginine and proline metabolism	up	9.49 × 10^−3^	1.75	Putrescine	C_4_H_12_N_2_	89.10725487
		Lysine degradation	down	4.08 × 10^−2^	1.33	Cadaverine	C_5_H_14_N_2_	103.122903
		Tryptophan metabolism	down	3.44 × 10^−2^	1.11	Indole	C_8_H_7_N	118.0650873
		Lysine biosynthesis	down	2.11 × 10^−6^	2.07	L-Saccharopine	C_11_H_20_N_2_O_6_	277.1390289
		Phenylalanine metabolism	down	2.18 × 10^−2^	5.22	Phenylacetaldehyde	C_8_H_8_O	121.0647704
		Arginine biosynthesis	down	5.84 × 10^−3^	2.74	L-Citrulline	C_6_H_13_N_3_O_3_	176.1028923
		Arginine and proline metabolism	up	8.30 × 10^−4^	11.94	Spermidine	C_7_H_19_N_3_	146.1651255
		Tryptophan metabolism	down	1.83 × 10^−2^	5.89	L-Kynurenine	C_10_H_12_N_2_O_3_	209.0919249
		Arginine and proline metabolism	down	9.08 × 10^−3^	4.49	Agmatine	C_5_H_14_N_4_	131.1290899
		Arginine and proline metabolism	up	3.60 × 10^−3^	2.51	1-Pyrroline-4-hydroxy-2-carboxylate	C_5_H_7_NO_3_	130.0498415
		Histidine metabolism	down	2.19 × 10^−2^	2.51	Urocanic acid	C_6_H_6_N_2_O_2_	139.0502099
		Lysine degradation	up	1.17 × 10^−2^	2.85	Deoxycarnitine	C_7_H_15_NO_2_	146.1175269
		Lysine degradation	down	1.27 × 10^−3^	2.68	L-Pipecolic acid	C_6_H_11_NO_2_	130.0862821
		Arginine and proline metabolism	down	2.11 × 10^−2^	1.28	N-Succinyl-L-glutamate	C_9_H_13_NO_7_	248.2077594
		Cysteine and methionine metabolism	up	2.01 × 10^−3^	3.80	Sulfite	SO_3_^2-^	83.084995
	Carbohydrate metabolism	Glyoxylate and dicarboxylate metabolism	down	2.99 × 10^−3^	4.62	L-threo-3-Methylaspartate	C_5_H_9_NO_4_	148.0603521
		Amino sugar and nucleotide sugar metabolism	down	1.80 × 10^−3^	1.72	N-Acetylneuraminate	C_11_H_19_NO_9_	310.1127173
		Amino sugar and nucleotide sugar metabolism	up	1.47 × 10^−2^	1.72	CDP-abequose	C_15_H_25_N_3_O_14_P_2_	534.3283127
		Pentose and glucuronate interconversions	down	3.45 × 10^−4^	16.41	CDP-ribitol	C_14_H_25_N_3_O_15_P_2_	538.3194126
		Amino sugar and nucleotide sugar metabolism	down	2.76 × 10^−3^	1.13	UDP-L-Ara4N	C_14_H_23_N_3_O_15_P_2_	536.3039803
		Amino sugar and nucleotide sugar metabolism	down	7.69 × 10^−4^	1.18	CDP-4-dehydro-3,6-dideoxy-D-glucose	C_15_H_23_N_3_O_14_P_2_	532.3185085
		Amino sugar and nucleotide sugar metabolism	down	4.66 × 10^−3^	2.99	N-Acetylmuramic acid 6-phosphate	C_11_H_20_NO_11_P	374.2531432
	Digestive system	Bile secretion	down	1.26 × 10^−2^	7.39	L-Carnitine	C_7_H_15_NO_3_	162.1123783
		Bile secretion	down	3.80 × 10^−4^	6.22	Acyclovir	C_8_H_11_N_5_O_3_	226.216309
	Lipid metabolism	Glycerophospholipid metabolism	up	1.57 × 10^−2^	7.49	sn-Glycero-3-phosphocholine	C_8_H_21_NO_6_P^+^	258.1097776
		Glycerophospholipid metabolism	up	4.97 × 10^−2^	2.77	LysoPC(16:1(9Z))	C_24_H_48_NO_7_P	494.3234809
		Glycerophospholipid metabolism	up	3.07 × 10^−2^	3.58	Glycerylphosphorylethanolamine	C_5_H_14_NO_6_P	216.062969
	Membrane transport	ABC transporters	up	1.58 × 10^−3^	1.84	Ferrichrome	C_27_H_42_FeN_9_O_12_	741.5250124
		ABC transporters	down	1.28 × 10^−3^	2.09	Mannopine	C_11_H_22_N_2_O_8_	311.3132994
	Metabolism of cofactors and vitamins	Porphyrin and chlorophyll metabolism	up	4.05 × 10^−3^	1.01	Biliverdin	C_33_H_34_N_4_O_6_	583.2537613
		Biotin metabolism	down	4.20 × 10^−3^	1.06	Biotin	C_10_H_16_N_2_O_3_S	245.0953355
		Thiamine metabolism	up	2.49 × 10^−3^	2.39	Thiamin triphosphate	C_12_H_19_N_4_O_10_P_3_S	506.2968678
		Nicotinate and nicotinamide metabolism	down	1.75 × 10^−2^	1.09	2,3-Dimethylmaleate	C_6_H_8_O_4_	145.1335282
	Metabolism of other amino acids	beta-Alanine metabolism	up	3.18 × 10^−3^	3.47	Pantothenic acid	C_9_H_17_NO_5_	220.1178677
**ESI−**	Amino acid metabolism	Valine, leucine and isoleucine degradation	down	3.54 × 10^−3^	2.84	L-VALINE	C_5_H_11_NO_2_	116.0717418
		Lysine biosynthesis	down	3.88 × 10^−3^	3.80	L-LYSINE	C_6_H_14_N_2_O_2_	145.098347
		Tryptophan metabolism	down	1.95 × 10^−2^	1.18	5-Hydroxyindoleacetic acid	C_10_H_9_NO_3_	190.0509535
		Lysine biosynthesis	down	7.28 × 10^−6^	1.02	L-Saccharopine	C_11_H_20_N_2_O_6_	275.1248028
		Alanine, aspartate and glutamate metabolism	up	2.46 × 10^−2^	1.09	Succinic acid semialdehyde	C_4_H_6_O_3_	101.0244019
	Biosynthesis of other secondary metabolites	Caffeine metabolism	down	5.30 × 10^−3^	1.07	7-Methylxanthosine	C_11_H_15_N_4_O_6_	298.2467506
	Carbohydrate metabolism	Citrate cycle (TCA cycle)	down	9.43 × 10^−3^	1.64	Succinic acid	C_4_H_6_O_4_	117.0193765
		Pentose and glucuronate interconversions	up	1.46 × 10^−3^	1.60	Ribitol	C_5_H_12_O_5_	151.0611315
		Glycolysis / Gluconeogenesis	up	2.63 × 10^−5^	2.23	D-Glycerate 3-phosphate	C_3_H_7_O_7_P	184.9855899
		Fructose and mannose metabolism	up	4.08 × 10^−2^	3.58	Mannitol	C_6_H_14_O_6_	181.0717565
		Amino sugar and nucleotide sugar metabolism	down	2.78 × 10^−3^	1.02	Uridine diphosphate-N-acetylglucosamine	C_17_H_27_N_3_O_17_P_2_	606.0741142
		C5-Branched dibasic acid metabolism	up	4.61 × 10^−2^	1.54	Itaconate, Itaconic acid	C_5_H_6_O_4_	129.0194034
		Ascorbate and aldarate metabolism	up	5.48 × 10^−3^	1.48	Threonic acid	C_4_H_8_O_5_	135.0299386
		Galactose metabolism	up	9.68 × 10^−3^	2.24	Tagatose	C_6_H_12_O_6_	179.0560745
		Pentose and glucuronate interconversions	down	1.10 × 10^−3^	2.43	CDP-ribitol	C_14_H_25_N_3_O_15_P_2_	536.3041792
		Pentose and glucuronate interconversions	down	3.65 × 10^−4^	11.32	CDP-ribitol	C_14_H_25_N_3_O_15_P_2_	536.3053925
		Amino sugar and nucleotide sugar metabolism	up	8.30 × 10^−4^	1.31	CMP-pseudaminic acid	C_22_H_34_N_5_O_15_P	638.5000382
	Energy metabolism	Oxidative phosphorylation	down	9.09 × 10^−3^	1.12	Pyrophosphate	P_2_O_7_^4−^	176.935889
	Lipid metabolism	Fatty acid biosynthesis	up	2.72 × 10^−3^	11.07	Caprylic acid	C_8_H_16_O_2_	143.1077396
		Fatty acid biosynthesis	up	7.01 × 10^−6^	15.37	Myristic acid	C_14_H_28_O_2_	227.2016801
		Fatty acid biosynthesis	down	3.75 × 10^−3^	10.03	Palmitoleic acid	C_16_H_30_O_2_	253.2172625
		Primary bile acid biosynthesis	up	2.73 × 10^−3^	5.33	Taurine	C_2_H_7_NO_3_S	124.0073745
		Fatty acid biosynthesis	up	7.76 × 10^−3^	5.94	Palmitic acid	C_16_H_32_O_2_	255.2329072
		alpha-Linolenic acid metabolism	down	1.33 × 10^−3^	5.58	Linolenic Acid	C_18_H_30_O_2_	277.2171486
		Linoleic acid metabolism	down	8.66 × 10^−3^	2.89	9-OxoODE	C_18_H_30_O_3_	293.2123043
		Biosynthesis of unsaturated fatty acids	down	6.24 × 10^−3^	1.21	Arachidic acid	C_20_H_40_O_2_	311.2954346
	Membrane transport	ABC transporters	up	6.64 × 10^−3^	4.01	Ferrichrome	C_27_H_42_FeN_9_O_12_	739.5113096
	Metabolism of cofactors and vitamins	Folate biosynthesis	down	3.34 × 10^−3^	4.26	2-Amino-4-hydroxy-6-(D-erythro-1,2,3-trihydroxypropyl)-7,8-dihydropteridine	C_9_H_13_N_5_O_4_	254.2205895
		Ubiquinone and other terpenoid-quinone biosynthesis	down	7.16 × 10^−4^	1.66	(1R,6R)-6-Hydroxy-2-succinylcyclohexa-2,4-diene-1-carboxylate	C_11_H_12_O_6_	239.2015915
		Folate biosynthesis	down	3.34 × 10^−2^	1.37	Neopterin	C_9_H_11_N_5_O_4_	252.2049325
	Metabolism of other amino acids	beta-Alanine metabolism	up	1.37 × 10^−2^	1.77	Pantothenic Acid (B_5_)	C_9_H_17_NO_5_	218.1033645
	Sensory system	Inflammatory mediator regulation of TRP channels	down	2.82 × 10^−3^	1.58	Icilin	C_16_H_13_N_3_O_4_	310.2830475

KEGG is the major public pathway-related database that includes not only genes but metabolites. ^†^ Pathway enrichment analysis identified significantly enriched metabolic pathways or signal transduction pathways in differential metabolites comparing with the whole background. The calculating formula is as follows: $P=1-\sum_{i=0}^{m-1}\frac{\left(\begin{array}{l} M\\ i \end{array}\right)\left(\begin{array}{l} N-M\\ n-i \end{array}\right)}{\left(\begin{array}{l} N\\ n \end{array}\right)}$. Here N is the number of all metabolites that with KEGG annotation, n is the number of differential metabolites in N, M is the number of all metabolites annotated to specific pathways, and m is number of differential metabolites in M. ^‡^ A variable importance in projection score of OPLS model was applied to rank the metabolites that best distinguished between two groups. ^§^ Means mass-to-charge ratio.

## Data Availability

Data is contained within the article or Supplementary Materials.

## Supplementary Materials

The following supporting information can be downloaded at: https://www.mdpi.com/article/10.3390/metabo12020158/s1, Table S1: The elution gradient of the mobile phase in LC-MS analysis.
