# Supplementary material for: Metabolic Changes in Larvae of Predator Chrysopa sinica Fed on Azadirachtin-Treated Plutella xylostella Larvae
Source: Metabolites. 2022 Feb 8;12(2):158. doi: 10.3390/metabo12020158 (PMC8876581; doi:10.3390/metabo12020158)
Supplement: Supplementary file 1 [file metabolites-12-00158-s001.zip › metabolites-1547007-supplementary.pdf]

Table S1. The elution gradient of the mobile phase in LC-MS analysis.

Time (min)	Flow rate (μL/min)	A%	B%
0	500	99	1
1.0	500	99	1
8.0	500	1	99
10.0	500	1	99
10.1	500	99	1
12.0	500	99	1

(The injection volume was 1 μL)
